# Surgical complications in neuromuscular scoliosis operated with posterior- only approach using pedicle screw fixation

**DOI:** 10.1186/1748-7161-4-11

**Published:** 2009-05-07

**Authors:** Hitesh N Modi, Seung-Woo Suh, Jae-Hyuk Yang, Jae Woo Cho, Jae-Young Hong, Surya Udai Singh, Sudeep Jain

**Affiliations:** 1 Scoliosis Research Institute, Department of Orthopedics, Korea University Guro Hospital, Seoul, Korea; 2 Rare Disease Institute, Department of Orthopedics, Korea University Guro Hospital, Seoul, Korea

## Abstract

**Background:**

There are no reports describing complications with posterior spinal fusion (PSF) with segmental spinal instrumentation (SSI) using pedicle screw fixation in patients with neuromuscular scoliosis.

**Methods:**

Fifty neuromuscular patients (18 cerebral palsy, 18 Duchenne muscular dystrophy, 8 spinal muscular atrophy and 6 others) were divided in two groups according to severity of curves; group I (< 90°) and group II (> 90°). All underwent PSF and SSI with pedicle screw fixation. There were no anterior procedures. Perioperative (within three months of surgery) and postoperative (after three months of surgery) complications were retrospectively reviewed.

**Results:**

There were fifty (37 perioperative, 13 postoperative) complications. Hemo/pneumothorax, pleural effusion, pulmonary edema requiring ICU care, complete spinal cord injury, deep wound infection and death were major complications; while atelectesis, pneumonia, mild pleural effusion, UTI, ileus, vomiting, gastritis, tingling sensation or radiating pain in lower limb, superficial infection and wound dehiscence were minor complications. Regarding perioperative complications, 34(68%) patients had at least one major or one minor complication. There were 16 patients with pulmonary, 14 with abdominal, 3 with wound related, 2 with neurological and 1 cardiovascular complications, respectively. There were two deaths, one due to cardiac arrest and other due to hypovolemic shock. Regarding postoperative complications 7 patients had coccygodynia, 3 had screw head prominence, 2 had bed sore and 1 had implant loosening, respectively. There was a significant relationship between age and increased intraoperative blood loss (p = 0.024). However it did not increased complications or need for ICU care. Similarly intraoperative blood loss > 3500 ml, severity of curve or need of pelvic fixation did not increase the complication rate or need for ICU. DMD patients had higher chances of coccygodynia postoperatively.

**Conclusion:**

Although posterior-only approach using pedicle screw fixation had good correction rate, complications were similar to previous reports. There were few unusual complications like coccygodynia.

## Background

Progressive spinal deformities are common in patients with neuromuscular disorders [1]. Typical neuromuscular scoliosis deformity involves the entire thoracic and lumbar spine with apex being near thoracolumbar junction, often creating pelvic obliquity and postural problems. Progressive deformity interferes with general health and well-being, ambulation, sitting-balance and wheelchair transmission. This disability ultimately leads to decubiti, costopelvic impingement pain, and worsening of pulmonary status [2]. Neuromuscular scoliosis is difficult to control with bracing, and, there is high risk of progression even after the skeletal maturity [3-5]. In addition, bracing may cause further pulmonary restrictions and interfere with feeding. General consensus amongst the deformity surgeons is to operate the patients with neuromuscular scoliosis for prevention of further progression of curve and, thereby, to prevent future complications [6-8]. Long-term studies have shown improvements in sitting position, quality of life, and lung function; improvement in spinal deformity; and improvement in pelvic obliquity [1,9-13]. Surgical treatment typically involves an instrumented fusion to pelvis with or without an anterior spinal release or fusion (ASF) [14]. Treatment of neuromuscular scoliosis with posterior-only pedicle screw instrumentation is a recent concept, which can obviate the need for ASF in idiopathic or neuromuscular scoliosis [15,16].

However, compared to idiopathic scoliosis, surgical treatment of neuromuscular scoliosis has typically been associated with a high complication rate. The reported rates range between 24% and 75% [9,17-19]. Various complications have been described following operative correction, mainly pulmonary (atelectasis, pneumo/hemothorax, pleural effusion), gastrointestinal (ileus), infectious (urinary tract and wound infections) and rarely neurologic [20]. There are also implant related complications, such as loosening and breakage of implants etc. All these complications have been described with combined ASF and PSF-SSI procedures. Others have described complications with anterior procedures [4,9,12,13,21-23]. Complications related with PSF have been described with various SSI systems [1,17-19]. However, there are no studies describing complications in neuromuscular scoliosis with PSF only using pedicle screw fixation. With increasing popularity of pedicle screws in scoliosis, it is worthwhile to study complications in neuromuscular group operated with these implants. The objective of this study was to study perioperative and postoperative complications in a group of patients with neuromuscular scoliosis treated by PSF and SSI only. In addition we attempted to identify some risk factors for a few complications.

## Methods

At our institute, between 2003 and 2005, 50 patients with neuromuscular scoliosis underwent surgical correction by a single spine surgeon (SWS) with PSF and SSI posterior-only pedicle screw fixation. None of the patient underwent anterior release or instrumentation procedure or other posterior procedures such as thoracoplasty or spinal osteotomy. Fusion was achieved using local bone graft mixed with allograft after thorough decortications of laminae. There were 21 patients with pelvic fixation to correct pelvic obliquity. Pelvic fixation was achieved with pedicle screws into the iliac wings directed towards the hip joint. These were connected with the main rod on either side using a connector. There were 32 male (64%) and 18 females (36%) with a mean age of 18.1 ± 8.2 years (range, 8–43 years). Diagnosis included 18 patients with cerebral palsy (CP), 18 patients with Duchenne muscular dystrophy (DMD), 8 patients with spinal muscular atrophy (SMA) and 6 patients with other diseases (2 polio, 2 neurofibromatosis, 1 multiple sclerosis, 1 Prader-Willi syndrome) (Table 1). We retrospectively reviewed the preoperative, intraoperative and postoperative records of all patients for correction and maintenance of correction in their spinal deformity, pelvic obliquity, thoracic kyphosis and lumbar lordosis; as well as any intraoperative or postoperative complications.

Complications that significantly affected the course of recovery, required ICU care more than 24 hours or endangered life or limb were considered major. All others were classified as minor. Hemo/pneumothorax, pleural effusion requiring chest tube insertion, pulmonary edema requiring ICU care, complete spinal cord injury, deep wound infection and death were the major complications; while atelectesis, pneumonia, mild pleural effusion, UTI, ileus, vomiting, gastritis, tingling sensation or radiating pain in lower limb, superficial infection and wound dehiscence were considered minor complications. Complications that occurred intra-operatively, during hospital stay or within three months after the operation were considered as perioperative complications [22]. Complications that occurred after three months postoperatively were considered as postoperative complications.

Both complications were reviewed separately. Spinal deformity preoperatively and postoperatively was measured by Cobb angle while pelvic obliquity was determined by drawing the line joining highest point of two iliac crests with respect to the horizontal line. Similarly, thoracic kyphosis was measured from upper end plate of T4 to lower endplate of T12, and lumbar lordosis was measured from upper endplate of L1 to upper endplate of S1 vertebra. Preoperatively there were two patient groups; curve < 90° (group I- mild and moderate scoliosis) and curve > 90° (group II- severe scoliosis). Additionally, we evaluated the correction of spinal deformity and pelvic obliquity between patients who had pelvic fixation and who did not, and between patients who had an age at surgery of 19 years or less and who had age of 20 years or more.

We analyzed the rate of minor and major complications according to the severity of the curve with chi-square test. We also determined the duration of operation and intraoperative blood loss to see if these were risk factors which are associated with complications or ICU need with chi-square test.

**Table 1:** Demographics of study group.

	**All patients (n)**	**Group I (n)**	**Group II (n)**
**Total no of patients**	50	30	20
**Cerebral Palsy**	18	11	7
**Duchenne muscular dystrophy**	18	12	6
**Spinal muscular atrophy**	8	3	5
**Others**	6	4	2
**Thoracic curves**	9	6	3
**Thoraco-lumbar curves**	30	19	11
**Lumbar curves**	11	5	6

**Average age (years ± SD)**	18.1 ± 8.2	15.9 ± 6.5	24.5 ± 8.5
**Average Follow-up (months ± SD)**	24.9 ± 8.8	25.3 ± 9.1	23.6 ± 9.1
**Average Cobb angle (degree ± SD)**	79.3 ± 30.3	58.1 ± 15.2	109.9 ± 17.7
**Average pelvic obliquity (degree ± SD)**	14.6 ± 9.4	11.7 ± 8.2	18.7 ± 9.8
**Average flexibility (%)**	29.7 ± 14.7	36.4 ± 14.2	19.9 ± 9

SD indicates standard deviation.

## Results

There were 30 patients with curve < 90° (group I) and 20 patients with curve > 90° (group II). Table 1 shows distribution of diagnosis, mean follow-up and curve pattern in each group. The mean preoperative curve and pelvic obliquity was 79.3 ± 30.3° (range, 40°–150°) and 14.6 ± 9.4° (range, 1°–39°) respectively. Preoperative flexibility of scoliosis curve was greater in group I 36.4% (range, 4°–32°) as compared to group II 20% (range, 9°–42°). This was statistically significant (p < 0.0001, unpaired t-test). The mean postoperative curve and pelvic obliquity improved to 31.3 ± 21.8° (63.1% correction) and 6.8 ± 6.3° (50% correction), respectively. At last follow-up they remained unchanged (scoliosis 32.6 ± 21.8° and pelvic obliquity 8.4 ± 8.3°). Comparing the correction rates between group I and group II, there was no significant difference either in scoliosis (p = 0.053, unpaired t-test) or pelvic obliquity (p = 0.363, unpaired t-test) (Table 2). Similarly preoperative, postoperative and final follow-up thoracic kyphosis was 25.7 ± 22.2°, 22.5 ± 10.7° and 23.1 ± 11.9°, and lumbar lordosis was (-)5.1 ± 31.1°, (-)26.4 ± 14.1° and (-)27.2 ± 14.8°, respectively. This demonstrated that thoracic kyphosis was well maintained and lumbar lordosis was improved and maintained at last follow-up (Table 3). We compared the radiological results between patients who had pelvic fixation and who did not, and found no significant difference in the correction of pelvic obliquity (p = 0.425, unpaired t-test). However, there was better correction rate in patients without pelvic fixation (p = 0.025, unpaired t-test) (Table 2). There was 58 ± 18% and 55 ± 30% correction in scoliosis and pelvic obliquity respectively in patients who had pelvic fixation; and 69 ± 15% and 47 ± 35% correction in patients who did not. Similarly, there was 64 ± 27% and 54 ± 25% correction in scoliosis and pelvic obliquity in patients who were 19 years or less and 53 ± 22% and 51 ± 44% correction for those 20 years or older at surgery. There was statistically no significant difference in the correction rate of scoliosis (p = 0.191, unpaired t-test) and pelvic obliquity (p= 0.438, unpaired t-test) according to age.

**Table 2:** Preoperative, postoperative and final follow-up Cobb's angle and pelvic obliquity with blood loss, operation time, hospital stay and ICU stay of study group.

	**No.**	**Cobb angle (degree ± SD)**			**Pelvic obliquity (degree ± SD)**			**Blood loss**	**Operation time**	**Hosp stay**	**ICU stay**
						
	**(n)**	**Preop**	**Postop**	**Final f-u**	**Preop**	**Postop**	**Final f-u**	**millilitres ± SD**	**min ± SD**	**days ± SD**	**(n)**
**All patients**	**48**	79.3 ± 30.3	31.3 ± 21.8	32.6 ± 21.8	14.6 ± 9.4	6.8 ± 6.3	8.4 ± 8.3	3221 ± 1711	361 ± 104	20.7 ± 6.7	13
**Group I**	**29**	58.1 ± 15.2	19.3 ± 13.4	20.6 ± 13	11.7 ± 8.2	4.8 ± 5.3	5.5 ± 6.8	2891 ± 1619	337 ± 92	19.2 ± 5.2	4
**Group II**	**19**	109.9 ± 17.7	48.7 ± 20.2	50.3 ± 20.1	18.7 ± 9.8	9.7 ± 6.7	12.7 ± 10	3715 ± 1767	398 ± 112	23.4 ± 8.2	9

**With pelvic fixation**	**21**	85.6 ± 26.9	38 ± 21.6	39.5 ± 21.1	23.7 ± 5.9	10.8 ± 7	14.5 ± 9.8	3547 ± 1837	403 ± 104	23.2 ± 7.3	7
**Without pelvic fixation**	**27**	74.2 ± 32.6	24.8 ± 21.2	27.1 ± 21.1	7.3 ± 4.2	3.5 ± 3.7	3.6 ± 4	2805 ± 1505	331 ± 98	18.9 ± 5.7	6

**Age < 20 years**	**35**	74.8 ± 27.2	26.8 ± 17.6	28.5 ± 17.4	14.1 ± 8.5	6.5 ± 6.3	7.4 ± 6.6	2884 ± 1656	356 ± 98	19.3 ± 5.1	8
**Age =/> 20 years**	**13**	90.4 ± 35.7	42.3 ± 27.7	43.4 ± 28.5	15.5 ± 11.8	7.5 ± 6.5	10.6 ± 13.1	4085 ± 1591	374 ± 121	24.7 ± 9.2	5

**Table 3:** Preoperative, postoperative and final follow-up thoracic kyphosis and lumbar lordosis in each group.

	**No.**	**Thoracic kyphosis (degree ± SD)**			**Lumbar lordosis (degree ± SD)**		
		
	**(n)**	**Preop**	**Postop**	**Final f-u**	**Preop**	**Postop**	**Final f-u**
**All patients**	**48**	25.7 ± 22.2	22.5 ± 10.7	23.1 ± 11.9	(-)5.1 ± 31.1	(-)26.4 ± 14.1	(-)27.2 ± 14.8
**Group I**	**29**	25.1 ± 21.9	25.5 ± 9.7	26.4 ± 10.2	(-)9.5 ± 22.7	(-)30.3 ± 13.1	(-)29.9 ± 13.3
**Group II**	**19**	29.5 ± 23.8	20.8 ± 12.1	22.2 ± 17.9	(-)1.9 ± 34.4	(-)23 ± 14.4	(-)23.7 ± 14.8

### Perioperative Complications (Table 4)

**Table 4:** Perioperative complications in patients.

**Major complications**	**All**	**Group 1**	**Group 2**	**Minor complications**	**All**	**Group 1**	**Group 2**
**Pulmonary Complications**							

Hemothorax/Pneumothorax	5	3	2	Pneumonia	4	2	2
Pleural effsion need Chest tube	1	1		Atelectesis	3	3	
Pulmonary edema need ICU care	2	1	1	Mild pleural effusion	1	1	

**Neurological Complications**							

Complete spinal cord injury	1	1		Tingling sensations or radiationg pain	1		1

**Abdominal complications**							

Pancreatitis				UTI	8	5	3
Superior messentric artery syndrome				Paralytic Ileus	3	2	1
Organic disease				Gastritis/vomitting	3	3	

**Cardiac complications**							

Arrythmia							
Cardiac arrest (Death)	1		1				

**Wound related complications**							

Deep wound infection	1		1	Superficaial infection	1		1
				Wound dehiscence	1	1	

**Death (other than cardiac arrest)**							

Hypovolemic shock	1	1					

Perioperative complications rates should be calculated from total operated numbers of patients (50).

There were total 37 perioperative complications in the study. There were 34 patients (68%) with at least one major or one minor complication. There were two (4%) deaths; one due to hypovolemic shock and one due to cardiac arrest.

There were 16 pulmonary complications (43%). Eight pulmonary complications were considered as major who required chest tube insertion or ICU care while the remaining 8 were considered minor and were treated conservatively (Table 4). Comparing pulmonary complications according to severity of curve patients who had curve < 90° had 5 major and 6 minor complications while patients who had curve > 90° had 3 major and 2 minor pulmonary complications. Thus, there was no relationship between pulmonary complication and curve severity (p = 0.589, chi-square test). The most common major pulmonary complication was hemothorax and most common minor complication was pneumonia. None of the patient required prolonged intubation or ICU care due to pulmonary complications.

There was only one patient (3%) who had a cardiac complication. This was sudden cardiac arrest due to arrhythmia. This patient died intra-operatively in spite of resuscitation due to associated hypovolemic shock. One more patient who had an estimated intraoperative blood loss of 7800 milliliters died in ICU immediate postoperatively due to severe hypovolemic shock. There was one major neurologic complication (complete spinal cord injury) and one minor (root injury) neurologic injury (5%). The patient who had complete spinal cord injury had paralysis due to mal-positioned screw in the spinal canal which was removed. However recovery did not occur. One patient with minor neurological injury had tingling sensations in both legs which recovered completely within six weeks of operation. There were 8 patients (22%) who developed urinary tract infection (UTI) which was thought to be due to catheterization. All were treated with oral antibiotics after urine culture and there were no sequels. There was no major abdominal complication but 6 patients had a minor complication (16%) other than UTI. Three patients had vomiting or gastritis, and three had abdominal pain due to paralytic ileus. All patients with paralytic ileus were treated by restricting oral-intake and intravenous infusion until they have good bowel sounds. They were started slowly on oral intake. There was no major abnormal on sonogram or abdominal CT scan. There was one patient with deep wound infection and two patients with minor wound complications (one wound dehiscence and one superficial wound infection). Deep wound infection was treated with repeated debridement with application of vacuum and the wound ultimately healed after one year and covered with flap coverage. Minor wound complications were treated with dressings only and drainage of hematoma and no further sequels. None of the patients required implant removal.

### Postoperative Complications (Table 5)

**Table 5:** Postoperative implant related complications in patients.

**Complications**	**No (n)**	**Percentage (%**
**Coccygodynia**	7	15
**Screw head prominence on convex side**	3	6
**Bed sore**	2	4
**Loosening of implants**	1	2

**Total**	13	27

Total numbers of complications were divided by 48 to find out complication rate (2 patients died perioperatively).

**Figure 1:**
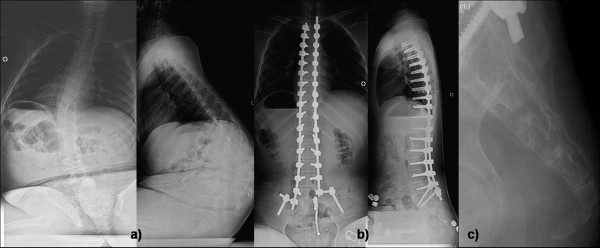
**shows a) preoperative AP and lateral sitting radiogram of a 13-year old boy with DMD with Cobb angle 50-degree operated by posterior only pedicle screw fixation developed good correction seen in b) postoperative AP and lateral sitting radiogram; however after six months he developed coccygodynia which exhibited c) coccyx subluxation on lateral coccyx radiogram**.

**Figure 2:**
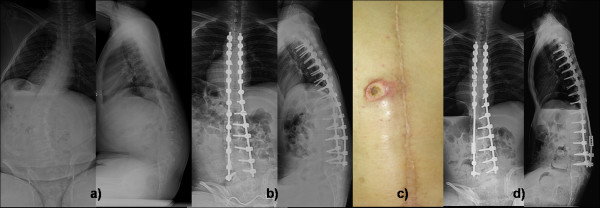
**shows a) preoperative AP and lateral radiogram of a 14-year old boy with DMD with Cobb angle of 44-degree operated by posterior only pedicle screw fixation**. b) postoperative AP and lateral radiogram shows good correction; however after one and half years he developed c) back sore due screw head irritation on convex side which was treated with removal of convex sided prominent three screws and connected with domino seen in d) AP and lateral radiogram after the procedure. Patient's sore was cured after the procedure.

These were mainly implant or fixation related complications. These were 7 patients (15%) had coccygodynia postoperatively, 6 patient had DMD and one had SMA. All patients had significant tenderness clinically over the coccyx with mild subluxation of the coccyx radiologically (Figure 1). All patients were treated conservatively with the use of soft cushions having round hole in the weight bearing area. Symptoms in 6 patients were decreased within six months. The remaining patient was a 13-year boy with DMD who ultimately required excision of subluxated coccyx. His symptom was relieved within six months after coccygeal excision. Two patients (4%) developed bedsore postoperatively; one was related with impingement of iliac screw with loosening which was treated with removal of screw; while another had gluteal sore which were treated with dressings and frequent position changes. Gluteal sores were considered to be due to neglected care of patients which was not due to implants or fixation related problems in the series. There were three patients (6%) who had problem due to screw head prominence on convex side; one had pain due to irritation from screw head and two had back sore due to impingement of screw head. All of them were treated with removal of at least three screws including most prominent screws and reconstructing the rod (Figure 2). After the revision operation all problems due to screw head resolved completely. There was one case with loosening of the screw from the distal level which was treated with reopening the site and fixing it again. There was no case with rod or screw breakage.

### Risk Factors

The mean operative time was 361 ± 104 min ranging from 180 min to 600 min. It was shorter in group I (337 ± 92 min, range 180–540 min) than group II (398 ± 112 min, range 195–600 min). This was statistically significant difference (p = 0.039, unpaired t-test). The mean estimate intraoperative blood loss (EBL) was 3221 ± 1711 ml (range, 750 ml to 7800 ml). It was less in group I (2891 ± 1619 ml, range 750–6000 ml) compared to group II (3715 ± 1767 ml, range 1000–7800 ml). This was not statistically significant (p = 0.095, unpaired t-test). Similarly patients who had pelvic fixation had more operative time than who did not have pelvic fixation (p = 0.019, unpaired t-test) but they did not have increased EBL (p = 0.130, unpaired t-test). There were 13 complications in patients who had pelvic fixation and 23 complications in those who did not. There was no statistically significant relation between pelvic fixation and complications (p = 0.192, chi-square test). Similarly there was no statistical relationship found between need of ICU care with pelvic fixation (p = 0.390, chi-square test). Similarly we have identified age as one of the risk factors. Comparing surgical time according to age, there was no relation between surgical time in patients who were 19 years or less than who were 20 years of age or older (0.590, unpaired t-test). While there was significantly increased intraoperative blood loss in older patients (0.024, unpaired t-test). However this does not establish a relationship of increased age with increased need of ICU care in older patients (p = 0.279, chi-square test). In spite of large numbers of complications, it is important to note that the magnitude or severity of complications were less.

## Discussion

Neuromuscular scoliosis remains a challenging problem for spinal deformity surgeons. Spine surgery in children with neuromuscular scoliosis aim to stabilize the spine, correct the deformity, halt deformity progression, improve sitting balance/function and prevent cardiopulmonary deterioration. Surgical care of such patients are often made worse by relatively poor nutrition and associated cardiopulmonary abnormalities. Complication rates between 24% and 75% have been reported after the surgical correction of neuromuscular scoliosis [9,17-19]. There are a few reports indicating perioperative complications (complications that occurred within three months of surgery) and long term implant related complications [21-25]. In addition most of the complications reported had combined ASF and PSF with SSI. However there is only one report of showing treatment of neuromuscular scoliosis with PSF with SSI using pedicle screws [26]. However, we could not find any article focusing on complications with PSF with SSI using pedicle screw instrumentation. In this study, we analyzed perioperative and postoperative complications that occurred in neuromuscular scoliosis operated with PSF with SSI using pedicle screw fixation.

Sarwahi et al [21] presented 24 (22%) with major and 25 (23%) patients with minor complications in their study of 111 neuromuscular scoliosis patients who were operated with combined ASF and PSF with SSI. The most common major complication was pulmonary (53%) and due to respiratory insufficiency. They required prolonged ICU care with ventilator support. They also noted that neuromuscular children had the lowest long-term survival. Janik et al [27] reported 28 patients (52%) with pulmonary complications of the total 51 complications in their series of 501 patients who were operated by anterior approach for various disorders. Our study found 16 of 37 (43%) pulmonary complications, and, if we consider only major complications, it will be only 8 (22%) of all complications.

Edler et al [28], in their study of 163 neuromuscular patients compared with 80 non-neuromuscular patients, noted that more than 65% of neuromuscular patients had blood loss > 50% of their estimated blood volume and, there was an almost seven times higher risk of loosing > 50% of their blood volume during scoliosis surgery in neuromuscular scoliosis. Shapiro and Sethna [29], in their review article, also reported higher blood loss in patients with neuromuscular scoliosis than idiopathic group mainly due to more number of vertebrae fused and combined approach. We agree to their findings that the mean blood loss in our study was 3221 ± 1711 ml and 20 patients had blood loss of 3500 ml or more and 16 of them developed at least one complication which suggested that there was a clear relation of developing complication in patients who had blood loss > 3500 ml (p = 0.087, chi-square test). In addition, 7 out of these 20 patients required ICU care which could not establish relationship of increased EBL and need for ICU support (p = 0.236, chi-square test). Literature search did not describe increase blood loss as a probable reason for high complication rates or need for ICU care.

Hod-Feins et al [30], compared outcomes in 95 idiopathic with 31 neuromuscular scoliosis, and found that postoperative complication parameters did not correlate to preoperative curve magnitude or number of vertebrae fused, and, noted that scoliosis surgery is safe even in severe deformities. Additionally, they noted that 20 patients who underwent spinal fusion using the combined approach had worse outcome than PSF or ASF alone. In present study we correlated complications between group I and group II.

We found that there were 24 (major and minor) complications in group I and 13 complications in group II. This showed no relationship between development of complication and curve severity (p = 0.236, chi-square test). In contrast, Sarwahi et al [21] reported postoperative complications in 12 out of 14 patients with deformities > 100° compared to in 37 out of 97 with curve < 100°. They concluded curve magnitude >100° was a risk factor for complications. Possibly, this was to an associated ASF while in this study we used posterior-only approach.

Wound infections are expected to be higher in neuromuscular scoliosis than idiopathic scoliosis or other spine problems [31-35]. Mohamad et al [22] has reported 18 (8%) wound infections in their study on perioperative complications in 175 neuromuscular scoliosis patients with 2 (1%) deep infections and 12 (7%) superficial infections. Similarly, Broom et al [18] reported 11% and McKeon et al [36] reported 12% infections. In our study we had only found 3 infections (8%) with mainly gram positive organisms such as coagulase positive or negative staphylococcus and methicillin resistant staphylococcus aureus and streptococcus. Sponseller et al [37] reported that gram negative organisms were isolated as commonly as gram positive organisms, eith the most common being coagulase negative Staphylococcus, Enterobacter, Enterococcus and Escherichia coli. The main difference is the posterior only approach in our study compared to combined approach in their study. In addition, they also identified severe cognitive impairment and use of allograft for the fusion as risk factors for the increased wound infections. However, we do not agree to that conclusion as we have used allograft in all our patients. One patient with poliomyelitis developed a deep wound infection while two patients with superficial infections had cerebral palsy.

The orthopaedic literature describes many abdominal complications after the surgery in neuromuscular scoliosis such as, pancreatitis, ileus, superior mesenteric artery syndrome, gall bladder disease and poor gastric motility [17,19,21-23,38,39]. However, in our study we did not experience any major abdominal complications. Most common abdominal complication was urinary tract infection (8 of 14 abdominal complications). Though the rate of abdominal complications was high (14 of 37, 38%), they all were minor without any long term effect. We feel this is due to the posterior-only approach. The other abdominal complications were gastritis, ileus and vomiting after the surgery which were controlled with restricting oral intake, intravenous fluids and anti-acidity medications. Ileus was considered as minor complication because it did not alter recovery in any patient [21].

Regarding cardiovascular complications, only one patient developed cardiac arrest due to arrhythmias and died intra-operatively. Regarding neurological complications, two (2 of 37, 5%) had developed neurological complications; one had a spinal cord injury due to a mal-positioned screw that compressed the spinal cord and was later removed. Unfortunately, there was no recovery. The other patient developed parathesias in both lower limbs which recovered completely within six weeks of surgery. This was thought to be due to spinal cord stretching as post operative CT scan did not show any root or spinal cord injury.

In our study, we did not have any implant related complications (except mal-positioned screw in the spinal cord) during perioperative period. Postoperatively there were 7 patients (7 of 48, 15%) who had coccygodynia; six out of them had DMD and one had SMA. Coccygodynia was typically associated with pain while sitting with significant tenderness over coccyx. Radiologically all patients exhibited mild subluxation of coccyx. All but one patients were treated successfully with conservative treatment aiming at relieve of pressure on coccyx. Only one patient with DMD who had severe subluxation required coccygeal excision (Figure 1). The reason for coccygodynia was believed to be due to increase sitting ability in those patients which ultimately increased pressure over coccygeal region. This is an unusual complication after correction with PSF and SSI with pedicle screw fixation, and it has not been described previously. Two patients (2 of 48, 4%) developed bed sore postoperatively; one was related with impingement of iliac screw with loosening which was treated with removal of screw while the other had a gluteal sore treated with dressings and frequent position change to avoid pressure sore. It was the only postoperative complication which we believed due to neglected care by care takers. There were three patients (3 of 48, 6%) who had problem due to screw head prominence on convex side [39]; one had pain due to irritation from screw head and three had back sore due to impingement of screw head. All of them were treated with removal of at least three screws, including the most prominent screws, and recontouring the rod (Figure 2). Back sores due to prominent screw heads healed completely after the revision surgery. Retrospectively we thought if we had used low profile screws, which have smaller screw head on convex side near the apex, it would have better avoided such complications. There was one case with loosening of the screw from the distal level which was treated with reopening the site and reinserting it. There were no cases of rod or screw breakage. It was interesting that all these implant related complications occurred after three months postoperatively which were included in a separate group.

## Conclusion

The correction of neuromuscular scoliosis with PSF and SSI using pedicle screw fixation has a complication rate similar to the other techniques. However the severity of complications were less than with an ASF [39]. However, few unusual complications, such as coccygodynia and back sores on convex side due to screw head prominence, should be remembered especially in neuromuscular scoliosis.

## Competing interests

The authors declare that they have no competing interests.

## Authors' contributions

HNM has contributed in conception and design and acquisition of data, analysis and interpretation of data, drafting the manuscript and revising it critically; SWS has contributed in conception and design of data, drafting the manuscript and given the final approval of manuscript; JYH contributed in acquisition of data, revising the manuscript critically and given the final approval; JHY has contributed in acquisition of data and analysis and interpretation of data; JWC has contributed in acquisition of data and analysis and interpretation of data; and SJ has contributed in revising the manuscript. All authors read and approved the final manuscript.
